# Human Gastroenteropancreatic Expression of Melatonin and Its Receptors MT1 and MT2

**DOI:** 10.1371/journal.pone.0120195

**Published:** 2015-03-30

**Authors:** Fanny Söderquist, Per M. Hellström, Janet L. Cunningham

**Affiliations:** 1 Department of Neuroscience, Psychiatry, Uppsala University, Uppsala, Sweden; 2 Department of Medical Sciences, Gastroenterology/Hepatology, Uppsala University, Uppsala, Sweden; University of Tennessee, UNITED STATES

## Abstract

**Background and Aim:**

The largest source of melatonin, according to animal studies, is the gastrointestinal (GI) tract but this is not yet thoroughly characterized in humans. This study aims to map the expression of melatonin and its two receptors in human GI tract and pancreas using microarray analysis and immunohistochemistry.

**Method:**

Gene expression data from normal intestine and pancreas and inflamed colon tissue due to ulcerative colitis were analyzed for expression of enzymes relevant for serotonin and melatonin production and their receptors. Sections from paraffin-embedded normal tissue from 42 individuals, representing the different parts of the GI tract (n=39) and pancreas (n=3) were studied with immunohistochemistry using antibodies with specificity for melatonin, MT_1_ and MT_2_ receptors and serotonin.

**Results:**

Enzymes needed for production of melatonin are expressed in both GI tract and pancreas tissue. Strong melatonin immunoreactivity (IR) was seen in enterochromaffin (EC) cells partially co-localized with serotonin IR. Melatonin IR was also seen in pancreas islets. MT_1_ and MT_2_ IR were both found in the intestinal epithelium, in the submucosal and myenteric plexus, and in vessels in the GI tract as well as in pancreatic islets. MT_1_ and MT_2_ IR was strongest in the epithelium of the large intestine. In the other cell types, both MT_2_ gene expression and IR were generally elevated compared to MT_1_. Strong MT_2_, IR was noted in EC cells but not MT_1_ IR. Changes in gene expression that may result in reduced levels of melatonin were seen in relation to inflammation.

**Conclusion:**

Widespread gastroenteropancreatic expression of melatonin and its receptors in the GI tract and pancreas is in agreement with the multiple roles ascribed to melatonin, which include regulation of gastrointestinal motility, epithelial permeability as well as enteropancreatic cross-talk with plausible impact on metabolic control.

## Introduction

Melatonin is well known as a pineal gland hormone that regulates sleep and circadian rhythm but there is also evidence for additional important regulatory functions [1]. Recent publications indicate that melatonin and its receptors regulate circulating glucose levels via insulin and glucagon secretion [2–4]. In the immune system, melatonin acts as an immunomodulator [5, 6] and both melatonin and its derivatives are powerful antioxidants, acting as scavengers of free radicals [7–9] for example protecting skin from UVR-induced damage [10]. Melatonin has been shown to promote cell survival in normal tissues [11–13], but to have oncostatic effects in various types of cancer [14–17]. While not widely known, it has previously been demonstrated in animal studies that the largest source of melatonin is the gastrointestinal (GI) mucosa [18]. The total amount of GI melatonin is estimated to be 400 times greater than that present in the pineal gland [18].

There are two receptors for melatonin, type 1A (MT_1_) and type 1B (MT_2_), both of which are G-protein coupled with high affinity in the nanomolar range [19]. Melatonin can also bind to retinoid related orphan nuclear hormone receptors (RZR/RORalfa)[20]. Subtypes of this nuclear receptor family display tissue specificity but their function is largely unknown [21]. There is a putative strong-affinity MT3 binding site that has been identified as a quinone reductase 2 [22], but its exact function, which involves NADP^+^/NADPH redox steps, remains to be determined [23].

In rats, mRNA transcripts of both MT_1_ and MT_2_ have been detected in the small intestine and colon [24, 25]. The highest expression of MT_1_ mRNA was detected in the subepithelial layers (muscularis externa and serosa) of the duodenum while the highest density of MT_2_ protein (using immunohistochemistry and western blot) was found in the colon, primarily in the smooth muscle layers [26]. One recent study has demonstrated MT_1_ immunoreactivity (IR) in human colon using immunohistochemistry (IHC) [27]. Another study on human duodenum showed melatonin, through MT_2_ receptors to be active on intracellular calcium storage [28]. Both rat MIN6 pseudoislets (beta cells) and human islets express mRNAs coding for MT_1_ and MT_2_ receptors, although human islet MT2 mRNA expression was low in this study [2]. Thus it seems that the presence of melatonin and its receptors in human GI tract and pancreas has not yet been fully characterized.

Melatonin in the GI tract appears to dampen intestinal motility [29, 30]. Levels of melatonin vary in relation to fasting and food intake. In pinealectomized rats, melatonin levels in the portal vein increase after tryptophan administration [31]. In humans and pigs, levels of melatonin do not follow a circadian rhythm but are elevated after food intake [32]. Short-term fasting in humans for two days reduces nocturnal concentrations of melatonin in serum [33]. In mice, fasting for 24 and 48 hours resulted in increased levels of melatonin in GI tissue, particularly in the stomach [34]. In rats, melatonin has been shown to release bicarbonate secretion and protect the mucosa [35]. Melatonin may also influence other hormones regulating hunger and satiety such as leptin and ghrelin [36, 37].

Our present study aims to localize the presence of melatonin and its receptors, MT_1_ and MT_2,_ at the cellular level in human GI tract and pancreas, using IHC and microarray expression analysis. This provides a basis for upcoming research on the regulatory function of melatonin in the upper and lower GI tract in man.

## Materials and Methods

### Ethics

The study was approved by the Regional Ethics Committee in Uppsala (Number: 2007/143, with decisions 2007-06-13 and 2012-09-14). Written informed consent was obtained for material collection but not this specific study on normal tissue as the data were analyzed anonymously with the purpose of method development and samples cannot be traced back to individuals. The Regional Ethics Committee waived the need for consent in this case in accordance with Swedish law.

### Microarray expression analysis

From the public gene expression data archive Gene Expression Omnibus (GEO) [38], raw data in the form of CEL-files from small intestinal epithelium GEO Series GSE9576 [39] and from human pancreas from the GEO Series GSE16515 [40] (n = 16) and GSE15471 [41] (n = 39) were extracted. The data in GSE9576 is based on RNA from immunolaser capture microdissected cells derived from three snap frozen histopathologically normal small intestinal epithelium specimens sectioned 10 μm thick and labeled with chromogranin A, where it is estimated that 25% of the captured cells are endocrine [39]. The data in GSE16515 and GSE15471 are histopathologically normal whole pancreas tissue from persons with pancreatic cancer [40, 41]. The normal samples from the above-mentioned datasets, all run on the same microarray platform “Affymetrix Human Genome U133 Plus 2.0 Array” (GEO Accession GPL570), were imported into Expression Console provided by Affymetrix (http://www.affymetrix.com). Normalization was performed using the robust multi-array average (RMA) method first suggested by Li and Wong in 2001[42, 43]. One sample “GSM388111”, from the GEO Series GSE15471, was excluded from further analysis as it failed the housekeeping gene control included on the array. Normalized Log2 expression signals were finally extracted from Expression Console including annotations.

Moreover the normal pancreatic islet samples from the GEO Series GSE3842[44] (n = 54), obtained from microarray analysis on the Affymetrix Human Gene 1.0 ST Array (GEO Accession GPL6244), were imported into Expression Console and further normalized as previously described. This data is based on RNA isolated islets from non-diabetic cadaver donors [44].

Further analysis was performed in the statistical computing language R (http://www.r-project.org), where the gene expression data for the key enzymes involved in production of melatonin from tryptophan, as well as receptors for serotonin and melatonin were extracted including annotations depicted in S1 and S2 Tables. Expression signals for parathyroid hormone (PTH) were used as a negative control. When the features could not be matched to the probe set ID, as in the case with the latter data set that is provided by a different microarray platform, matching was done based on Gene Symbol. The expression values for each feature were finally averaged for each GEO series separately and the results were visualized in separate plots.

The Linear Models for Microarray Analysis (*limma*) R package on GEO2R (http://www.ncbi.nlm.nih.gov/geo/info/geo2r.html) was used in order to find differentially expressed between colon biopsies from UC patients (inflamed) and healthy controls (not inflamed) from the GEO series GSE11223 [45]. A difference in gene expression exceeding log2 fold change (logFC) ± 0.6 was regarded as an indication of at least 50% up- or down-regulation. Only the samples obtained from the ascending, descending and sigmoid part of the colon were used. A box plot of the value distribution was constructed in order to check that the data is median-centered across the samples, which generally suggests that the data is normalized and cross-comparable. Statistical analysis of differences in expression levels were performed within data sets using two-tailed unpaired t test.

### Histology

#### Control tissues

Human tissue, representing different parts of the GI tract, was obtained from resection tissue margins removed from adult patients during surgery for malignancies; the margins were located at least 2 cm away from the neoplasm. Only tissue with normal macro- and microscopic morphological appearance were included in the study. The identity and their clinical data of the patients are not known and are not included in the analysis. Material from the GI tract of 39 individuals and from the pancreas of 3 individuals. (17 purchased from Asterand, Detroit, MI, USA and 25 from the Department of Pathology, Uppsala University Hospital, Uppsala, Sweden). Positive controls for MT_1_ and MT_2_ were performed on tissues from skin (2) (obtained from the Department of Immunology, Genetics and Pathology, Uppsala University Hospital) were studied.

#### Immunohistochemistry and microscopic assessments

Tissue specimens were fixed in 4% buffered formalin for 1–2 days, dehydrated, and embedded in paraffin wax. Sections, 4 μm thick, were attached to positively charged glass superfrost slides (Menzel-Gläser, Braunschweig, Germany). Sections were deparaffinized in xylene and rehydrated using decreasing concentrations of ethanol to distilled water. Antigen retrieval was performed using pressure cooker treatment for 10 minutes in citrate buffer pH 6. For MT_1_, sections were incubated for 30 minutes in 0.1% H_2_O_2_ to quench endogenous peroxidase, washed in phosphate-buffered saline (PBS), and then incubated with normal horse serum diluted 1:5 for 30 minutes. (Vector Laboratories, Burlingame, CA, USA). The sections were then incubated overnight at 4°C with a polyclonal goat anti-human MT_1_ antibody (Anti-MTR-1A, (N-20), sc13179, Santa Cruz Biotechnology, Dallas, Texas, USA diluted 1:500). Sections were washed thrice in PBS and then incubated with biotinylated horse anti-goat antibody (BA-9500, Vector Laboratories). Thereafter, sections were incubated for 30 minutes with avidin-biotin—horseradish peroxidase (PK-6100, Vectastain Elite ABC kit, Vector Laboratories). Rabbit anti-melatonin (0100–0203 AbD Serotec, Kidlington, UK, diluted 1/500), rabbit anti-melatonin receptor 1B (ABIN122307, Antibodies-online GmbH, Aachen, Germany diluted 1:100) and monoclonal mouse anti-serotonin (Clone 5HT-H209 DAKO Sweden AB, Stockholm, Sweden, diluted 1/100) antibodies were diluted in PBS with 1% bovine serum albumin (BSA). The DAKO EnVision™ systems for rabbit and for mouse (DAKO Sweden AB, Stockholm, Sweden) were used for antibody detection according to the manufacturer’s instructions. Diaminobenzidine was used as chromogen. For visualization of nuclei, the sections were counterstained with Mayer’s haematoxylin.

The specimens were altogether from 42 patients, 12 from stomach (fundus (n = 1), corpus (n = 5), antrum (n = 3) and pylorus (n = 3)), 11 from small intestine (duodenum (n = 3) and ileum (n = 8)), 3 from appendix, 13 from large intestine (cecum (n = 1), colon (n = 8), rectum (n = 4)) and 3 from pancreas. All specimens were coded and examined by two independent observers (FS and JLC) on two separate occasions. Staining intensity was classified as negative, weak, medium or strong for MT_1_ and MT_2_ and the cellular and intracellular localization was documented. The evaluators were in agreement in 92% of the cases. When disagreement occurred sections were reexamined and consensus reached. Sections from skin were used as a positive control for MT_1_ and MT_2_. Differences between melatonin expression in the different parts of the gastrointestinal tract was tested with the Kruskal-Wallis Test and the Mann Whitney was used as a post-hoc test.

#### Double immunofluorescence

For double immunofluorescence staining, sections were prepared as described above and then incubated overnight at 4°C with a cocktail of two primary antibodies (dilutions were as follows: anti-MTR-1A at 1:50, anti-melatonin receptor 1B at 1:50, anti-serotonin 1:50, anti-melatonin at 1:100). Before application of the antibody cocktail, the sections were incubated with a non-immune serum from the animal species producing the secondary antibody, diluted 1:10 in PBS with 1% BSA. The secondary antibodies used were: tetramethyl rhodamine isothiocyanate (TRITC)-conjugated rabbit anti-goat TRITC (Alexa 555 A 21431) fluorescein isothiocyanate (FITC)-conjugated goat anti-mouse (Alexa 488 A 11001) TRITC-conjugated goat anti-rabbit (Alexa 555 A 21428) and FITC-conjugated rabbit anti-mouse (Alexa 488 A 11059) all from Life Technologies Europe BV (Stockholm, Sweden). The incubation time for the secondary antisera was 30 minutes at room temperature. The sections were examined in a Zeiss 510 confocal microscope and photographed with an AxioCam camera employing ZEN 2012 imaging software (Carl Zeiss AB, Stockholm, Sweden) and a 40X plan-apochromat objective. Co-localization studies were performed on tissue sections from the pylorus (n = 2) and ileum (n = 2).

#### Antibody specificity tests

A neutralization test was performed for melatonin antibodies using saturated (0.1 mg/mL) solutions of melatonin and serotonin (0.1 mg/mL) in PBS with 1% BSA. Specificity tests were performed for the MT_1_ and MT_2_ antibodies using IHC. Control immunostaining included omission of the primary antisera, and replacement of the primary antibody by non-immune serum at the same dilution as the primary antibody in question and in the same diluent. A neutralization test was conducted by means of 24-hour incubation of primary antiserum with the relevant antigen (sc-13179P, Santa Cruz Biotechnology, Dallas, Texas, USA and SP4391CP, 10 nmol antigen per mL, diluted 1:100 antibody solution) before application to the sections.

## Results

### Microarray expression analysis

**Fig 1:**
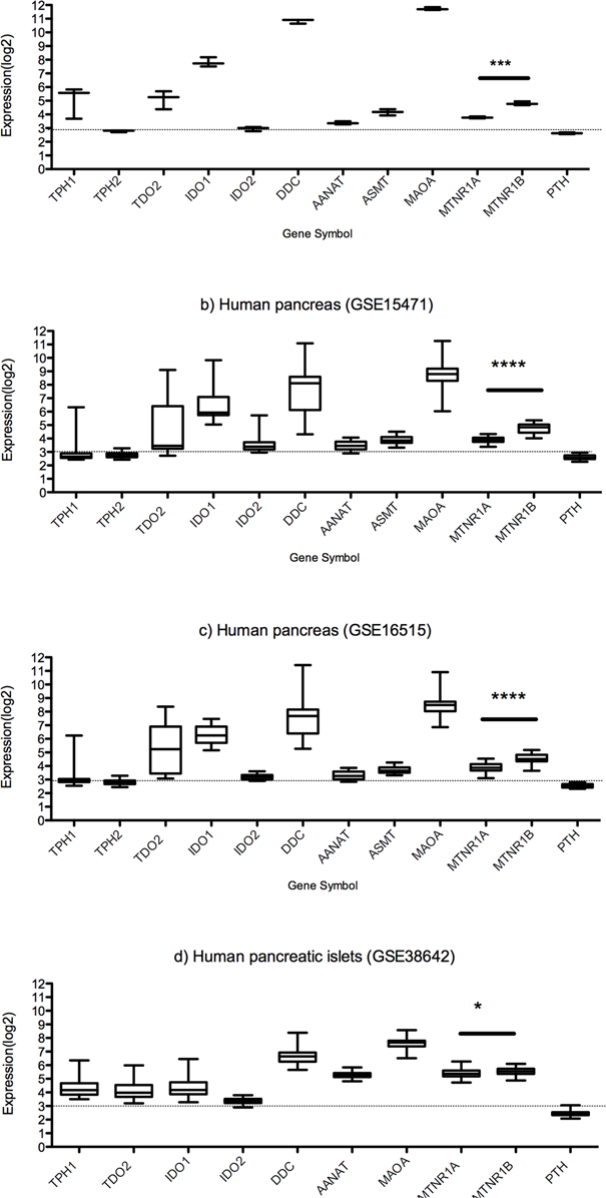
Normalized gastropancreatic gene expression data (log2) for key enzymes in tryptophan serotonin and melatonin metabolism and receptors for melatonin and serotonin. a) Human small intestinal epithelium (GSE9576) b) Human pancreas (GSE15471) c) Human pancreas (GSE16515) d) Human pancreatic islets (GSE38642). MT_2_ expression levels exceed MT_1_ levels in small intestine, whole pancreas and islets. (* p<0.05, *** p<0.001, **** p<0.0001).

Gene expression data for key enzymes in tryptophan metabolism as well as for serotonin and melatonin receptors were analyzed in data from small intestine epithelium enriched for enterochomaffin cells, whole pancreas and pancreatic islets (see S1 and S2 Tables). The data distribution, visualized with a histogram, showed that expression (log2) < 3 provided an indication of very low or absent expression. Additionally, RNA levels of PTH were used as a negative control (see Fig. 1).

In the samples from small intestine, tryptophan hydroxylase 1 (TPH1) and DOPA decarboxylase (DDC) were expressed and DCC levels were very high (log2expression >10). Gene expression of the enzymes needed for the production of melatonin from serotonin, arylalkylamine N-acetyltransferase (AANAT) and acetylserotonin O-methyltransferase (ASMT or HIOMT) were present. Both of the melatonin receptors were expressed with MT_2_ expression higher than of MT_1_ (p = 0.0003) in small intestinal mucosa, as found also with IHC analysis (see below).

Two sets of data from normal whole pancreas were analyzed with nearly identical results. TPH1 expression was generally low but there were some individuals with elevated levels, while DDC expression was high in all cases. AANAT and ASMT were present but at low levels. MTNR1A (MT_1_) and MTNR2B (MT_2_) were expressed at levels comparable to those of the small intestine. The levels of MTNR2B exceeded levels of MTNR1A in both data sets (p<0.0001).

In pancreatic islets, the expression patterns for TPH1 and DDC resembled those of whole pancreas. AANAT, MTNR1A and MTNR2B were all expressed at similar levels, data for TPH2 and ASMT was not available in this set. Levels of MTNR2B were higher than for MTNR1A (p = 0.02).

Tryptophan hydroxylase 2 (TPH2) is a neuronal isozyme of TPH1. The expression of TPH2 was very low or absent in the small intestine and whole pancreas. Tryptophan 2,3-dioxygenase (TDO2), indolamine 2,3-dioxygenase 1 and 2 (IDO1 and IDO2) catalyze the first and rate-limiting step of the kynurenine pathway. TDO2 was expressed in both the small intestine (mucosal cells enriched for endocrine cells) and in pancreatic islets at the same level as TPH1, while IDO1 levels exceeded TPH1 levels in small intestine (p = 0.02) and were similar as TPH1 in pancreatic islets. IDO2 levels were low in the tissues studied.

When inflamed tissue from ulcerative colitis was compared to controls regarding gene expression of enzymes for melatonin synthesis and its receptors, no difference in gene expression exceeding 1.5X (log2 fold change ± 0.6) was observed (see Table 1). The largest difference observed in the gene set was for TDO2 (logFC 0.22, p< 0.01), IDO1 (logFC 0.38, p = 0.0004) and DDC (logFC -0.19, p = 0.000004).

**Table 1:** Data from GEO series GSE11223 was analyzed using the limma R package on GEO2R in order to find differentially expressed genes between colon biopsies from UC patients (inflamed) and healthy controls (not inflamed).

Gene symbol	ID	logFC	P-value	Adjusted P-value
TPH1	3964	0,01	0,744000	0,824000
TPH2	39781	0,07	0,186000	0,304000
TPH2	39781	0,07	0,186000	0,304000
**TDO2**	**29668**	**0,22**	**0,001020**	**0,006090** *
**IDO1**	**13847**	**0,38**	**0,000001**	0,000042***
**DDC**	**4223**	**-0,19**	**0,000000**	0,000004***
AANAT	13791	0,00	0,604000	0,715000
ASMT	18325	0,02	0,103000	0,195000
MTNR1A	27435	0,00	0,002580	0,012300
MTNR1B	40793	-0,01	0,449000	0,580000
HTR1A	20428	0,04	0,886000	0,926000
HTR1B	16252	0,02	0,864000	0,911000
HTR1D	12629	0,04	0,025100	0,068000
HTR1E	31707	0,04	0,232000	0,358000
HTR1E	4728	0,00	0,001100	0,006430*
HTR1F	2587	0,07	0,039800	0,095500
HTR2A	21676	0,02	0,844000	0,897000
HTR2B	38243	0,00	0,169000	0,283000
HTR2C	25384	0,01	0,132000	0,234000
HTR3A	1107	-0,02	0,816000	0,878000
HTR3A	37312	0,04	0,874000	0,918000
HTR3B	11736	0,03	0,012400	0,040100
HTR3C	30062	-0,03	0,016100	0,048800
HTR3D	22905	0,02	0,130000	0,233000
HTR3E	9603	0,02	0,100000	0,191000
HTR4	13456	0,01	0,164000	0,276000
HTR4	636	-0,02	0,095200	0,184000
HTR5A	6663	0,06	0,306000	0,439000
HTR5A	21557	-0,02	0,559000	0,677000
HTR6	36124	0,06	0,008660	0,030800
HTR7	19544	0,03	0,160000	0,271000
HTR7P1	33553	0,03	0,000134	0,001280*

Genes with logFC> 0.1 are marked in bold typeface and significance is indicated as follows:

*p<0.01,

**p<0.005,

***p<0.0001.

### Histology

Immunoreactive cells for melatonin and its receptors were found throughout the GI tract and pancreas. Staining with melatonin, MT_1_ and MT_2_ antibodies were completely blocked by preincubation with the respective antigens. Preincubation with serotonin partially blocked the melatonin immunostaining.

**Fig 2:**
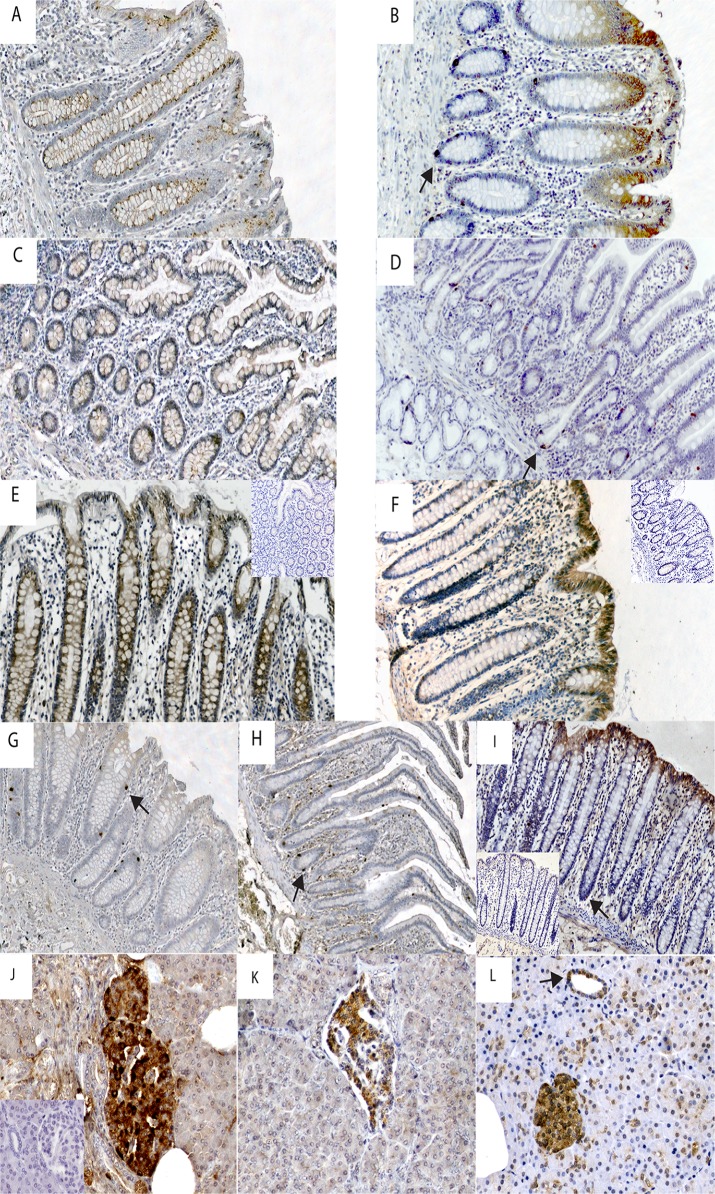
Immunohistochemical staining of gastrointestinal tract and pancreas tissue with antibodies against melatonin and receptors MT_1_ and MT_2_. A) Strong MT_1_ receptor immunoreactivity (IR) in epithelium in pyloric mucosa. B) Strong MT_2_ receptor IR in epithelium and endocrine cell (arrow) in pyloric mucosa. C) Weak MT_1_ receptor IR in epithelium in ileal mucosa. D) MT_2_ receptor IR is negative in epithelial cells but strong in endocrine cells (arrow) in duodenal mucosa E) Strong MT_1_ receptor IR in epithelium of colon mucosa. Insert shows neutralization test for MT_1_. F) Strong MT_2_ receptor IR in epithelium and endocrine cells (arrow) in colon mucosa. Insert shows neutralization test for MT_2_. G) Strong melatonin IR in endocrine cells in pyloric mucosa. H) Strong melatonin IR in endocrine cells in ileal mucosa. I) Strong melatonin IR in epithelial cells and endocrine cells in colon mucosa. Insert shows neutralization test for melatonin. A-I: Magnification 100X. J) Strong melatonin IR in endocrine cells in pancreatic islets. Inset shows negative serotonin IR. K) Strong MT_1_ receptor IR in endocrine cells in pancreatic islets. L) Strong MT_2_ receptor IR in endocrine cells in pancreatic islets and pancreatic ducts. (J-K: Magnification 200X).

**Fig 3:**
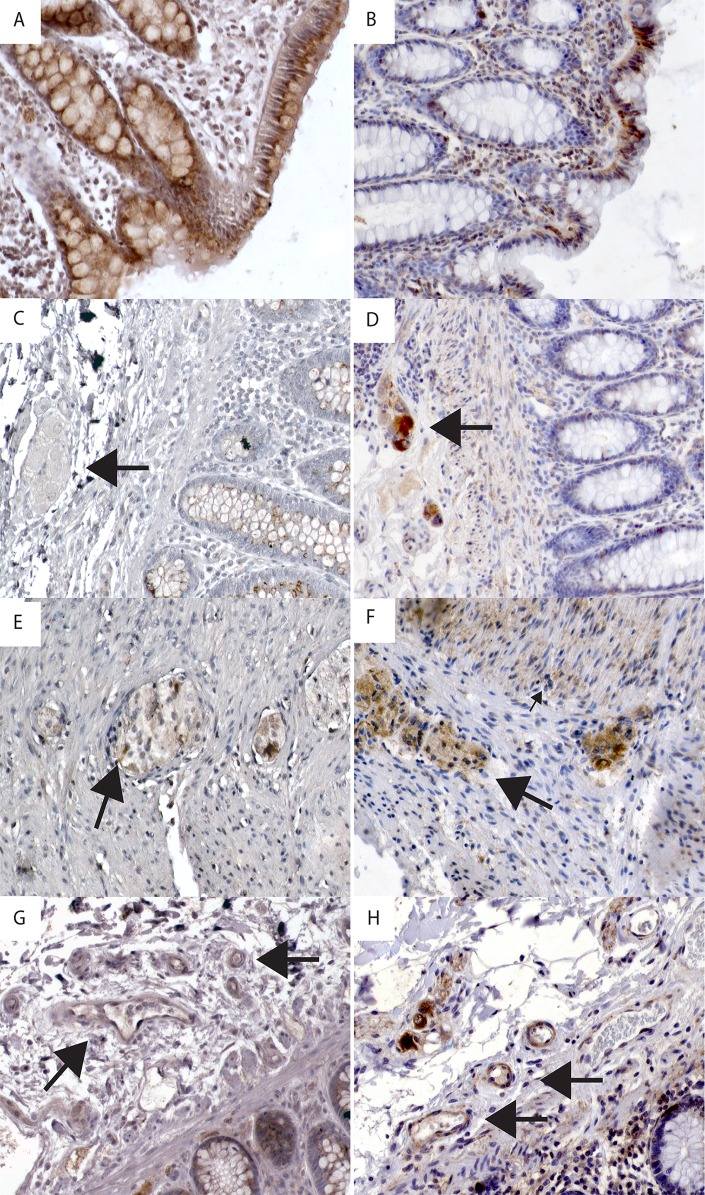
Immunohistochemical staining of melatonin receptors in specific cell types. A) Positive melatonin receptor 1 (MT_1_) immunoreactivity (IR) in epithelial cells. B) Melatonin receptor 2 (MT_2_) IR in epithelial cells. C) Arrow indicates negative MT_1_ IR in the submucosal plexus. D) Arrow indicates positive MT_2_ IR in the submucosal plexus. E) Arrow indicates a cell showing positive MT_1_ IR in the myenteric plexus; muscle cells are negative. F) Large arrow indicates positive MT_2_ IR in the myenteric plexus, small arrow indicates positive IR in muscle tissue. G) Large arrow indicates weak MT_1_ IR in the endothelium of arterioles and venules. H) Large arrow indicates MT_2_ IR in the endothelium and smooth muscle of arterioles and venules. Magnification 200X.

Intense melatonin IR was seen in enterochromaffin (EC) cells (See Table 2). In the stomach, five sections displayed plentiful EC cells with distinct serotonin and melatonin IR. Sections negative for serotonin also lacked melatonin. Throughout the remaining GI-tract numerous EC cells with distinct IR (see Fig. 2G-I) were identified. Co-localization studies showed that melatonin IR was diffusely spread in the cytoplasm and only partially co-localized with serotonin IR (see Fig. 3A-C). In a small fraction of EC cells, melatonin IR was much stronger than serotonin IR. Diffuse melatonin IR was found in the absence of serotonin IR in the cytoplasm of surface columnar epithelium in both mucus-producing cells (mucous neck cells and goblet cells) and columnar enterocytes with weak intensity in few cases from the stomach, and small intestine. In contrast, epithelial expression was stronger in all but one case from the large intestine (see Fig. 2I). Melatonin IR was found in the cytoplasm of mononuclear immune cells in the lamina propria. Finally, strong melatonin IR was discovered in a majority of the endocrine cells in the pancreatic islets (3/3) (see Fig. 2J) while serotonin IR was absent.

**Table 2:** Localization of melatonin (Mel) and receptors (MT_1_ and MT_2_) in human gastrointestinal tract and pancreas assessed using immunohistochemistry.

	Antibody	IR	Tissue					Significance^3^
			Stomach (a)	Small intestine (b)	Appendix (b)	Large intestine (c)	Pancreas	
**Number of cases**			12	11	3	13	3	
**Positive (%)**	Mel		42	100	100	100	100	
	MT1		58	64	100	100	33	
	MT2		100	100	100	100	100	
**Epithelial cells (n)**	Mel	Negative	9	7	3	1	0	
		Weak	3	4	0	0	3	
		Strong	0	0	0	12	0	c>a,b***
	MT1	Negative	5	4	0	0	0	
		Weak	5	7	3	0	1	
		Medium	2	0	0	4	0	
		Strong	0	0	0	9	0	c>a,b***
	MT2	Negative	7	4	0	0	3	
		Weak	0	7	2	1	0	
		Medium	5	0	1	3	0	
		Strong	0	0	0	9	0	c>a,b***
**Endocrine cells (n)**	Mel	Negative	7^(2)^	0	0	0	0	
		Weak	1	0	0	0	0	
		Strong	4	11	3	13	3	c,b>a***
	MT1	Negative	12	11	3	13		
		Medium	0	0	0	0	1	ns
	MT2	Negative	7	0	0	0	0	
		Medium	1	0	0	0	0	
		Strong	4	11	3	13	3	c,b>a***
**Submucosal plexus (n)** ^**1**^	MT1	Negative	10	9	2	5	na	c>a,b*
		Weak	0	1	1	8		
	MT2	Weak	3	3	2	3	na	
		Medium	7	7	1	5		
		Strong	0	0	0	5		ns
**Myenteric plexus (n)** ^**1**^	MT1	Negative	5	4	1	4	na	
		Weak	0	5	2	9		c>a*
	MT2	Weak	2	4	2	1	na	
		Medium	3	6	1	8		
		Strong	0	0	0	4		ns
**Vasculature (n)** ^**1**^	MT1	Negative	10	10	3	1	3	
		Weak	0	0	0	12	0	c>a,b**
	MT2	Weak	4	8	1	3	2	
		Medium	4	2	2	8	1	
		Strong	2	0	0	2	0	ns

Melatonin, MT_1_ and MT_2_ IR was strongest in the large intestine epithelium. At all levels of the gastrointestinal tract, the percentage of MT_2_-positive epithelial cells varied greatly between cases (from 5% to >75). Melatonin and MT_2_ and immunoreactivity (IR) is found in endocrine cells in the gut and pancreas. These cells are most plentiful in the small and large intestine. Melatonin IR was not assessed for plexus and vasculature. Nerve and vascular tissue showed both MT_1_ and MT_2_ IR although this was more frequent and stronger for MT_2._ Numbers of individuals where tissues were available for assessment is indicated under N. Percentage of individuals where tissue showed IR and the tissue type where IR was found is indicated (n). “na”: not applicable

^1^Shown for cases where sufficient tissue was available for evaluation.

^2^Serotonin IR is also negative for these sections.

^3^Differences between expression in stomach (a), small intestine and appendix (b) and large intestine (c). Significance tested with the Kruskal-Wallis Test and the Mann Whitney U Test was used as a post-hoc test,

*p<0.05,

**p<0.005,

***p<0.0001,

ns = not significant.

**Fig 4:**
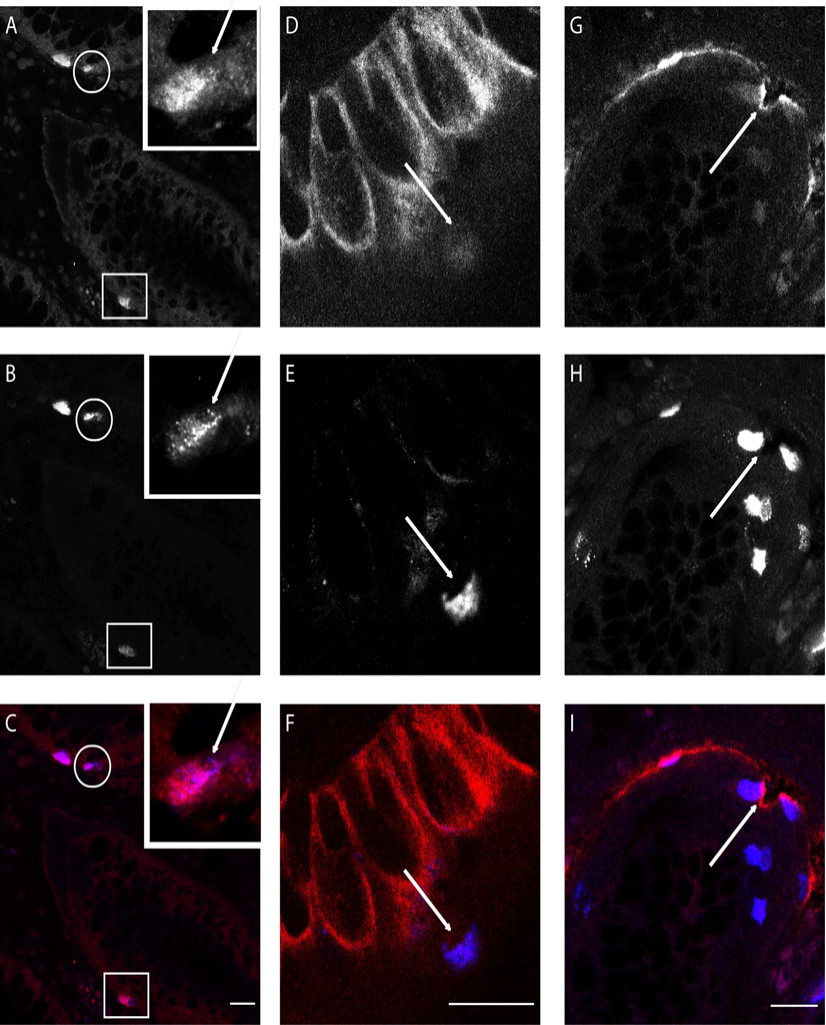
Confocal images of double immunofluorescence staining of crypts of Lieberkühn in ileum mucosa. **A**: Melatonin immunoreactive (IR) cells. **B**: Serotonin IR cells. **C**: Merge **A-C**; Inset: magnification of cell in circle. Arrow indicates structures positive for serotonin but not melatonin. Square indicates cell where melatonin IR is strong compared to serotonin IR. **D**: Melatonin receptor MT_1_ IR in crypt epithelium. **E**: Serotonin IR cell. **F**: Merge **D-F**; Arrow indicates serotonin IR cell negative for melatonin receptor MT_1_. **G**: Melatonin receptor MT_2_ IR. **H**: Serotonin IR cells. **I**: Merge **GI**: Arrow indicates serotonin IR cell positive for melatonin receptor MT_2._ White bar indicates 20 μm.

MT_1_ IR was found primarily in the cytoplasm of epithelium in both mucus-producing cells (mucous neck cells and goblet cells) and columnar enterocytes (see Fig. 2A and Table 2). Weak to medium MT_1_ IR was found in the stomach (7/12), small intestine (7/11) and appendix (3/3) (see Fig. 2A, C and E). The most intense MT_1_ IR was observed in the epithelium of the large intestine where all sections were positive. No relationship between EC cells and MT_1_ IR was seen. Weak MT_1_ receptor IR was rarely found in the submucosal plexus of the small intestine (1/10) or the appendix (1/3), and was found more often in the large intestine (8/13). Weak MT_1_ receptor IR was noted in the myenteric plexus in the small intestine (5/9), in the appendix (2/3) and in the large intestine (9/13) (see Fig. 3E). Smooth muscle cells were negative for the MT_1_ IR. In vascular structures, weak MT_1_ IR was seen in endothelial cells in the distal parts of the GI tract; the appendix (3/3) and the large intestine (12/13) (see Fig. 3G). EC cells were negative for MT_1_ IR (see Fig. 4D-F). In pancreatic tissue, MT_1_ IR varied. In one case, strong IR was found in a subset of cells in pancreatic islets and weaker IR was seen in pancreatic acini (see Fig. 2K). The remaining two cases were negative.

MT_2_ IR was most prominent in the epithelium, localized to nuclei as well as cytoplasm of enterocytes throughout the GI tract, with the strongest IR in the large intestine (see Fig. 3B and Table 2). Additionally, all sections from the small and large intestine and 5 of 12 gastric sections showed cytoplasmic MT_2_ staining of endocrine cells in the crypts (see Fig. 2B, D and F). Negative gastric sections were also negative for serotonin IR. Co-localization studies verified MT_2_ IR and serotonin IR, a marker for EC cells, in the same cells but in different cytoplasmic compartments (see Fig. 4G-I). Cells positive for serotonin IR but negative for MT_2_ receptor IR were also noted. Weak MT_2_ staining was present in intestinal smooth muscle cells in all sections. Cytoplasmic MT_2_ IR was noted in both the submucosal and myenteric plexuses throughout the GI tract (see Fig. 3D and F). A small number of immune cells in the lamina propria also displayed nuclear and cytoplasmic MT_2_ receptor IR (see Fig. 3B). MT_2_ IR was observed in a subset of cells in the vascular smooth muscle cells of all tissues studied (see Fig. 3H). Strong MT_2_ IR was found in a subset of cells in the endocrine pancreas (3/3) and in cells lining the pancreatic ducts, while IR in acinar cells was not seen (see Fig. 2L). Table 2 summarizes the findings for MT_1_ and MT_2_ in different cell types.

## Discussion

Animal studies have established the presence of melatonin in the GI tract. Little data on human GI tract and pancreas is available. A limitation of our study and a general problem for many studies of human tissue is that histologically normal tissue is usually not obtained from healthy persons. In this study, tissue was obtained during surgery after standardized fasting or from cadavers, which may influence the expression of hormones and their receptors.

In the tissue, l-tryptophan is converted to serotonin with enzymes TPH1 (or TPH2 in neuronal cells) and DDC. Melatonin is then synthesized from serotonin through two enzymatic steps, AANAT and ASMT. It is, therefore, not surprising that melatonin was found throughout the gut. Our analysis of gene expression data show that AANAT and ASMT are expressed in the small intestine epithelial cells enriched for endocrine cells. The Human Protein Atlas also verified positive IHC staining for AANAT throughout the GI tract (http://www.proteinatlas.org/ENSG00000129673-AANAT/tissue) [46]. Interestingly, AANAT staining is located in the glandular cells of the mucosa and not restricted to endocrine cells. The lower expression levels for AANAT compared with ASMT (p = 0.0055) is in agreement with the indication that AANAT was the rate-limiting enzyme for melatonin production and ASMT catalyzing the final reaction in the synthesis of melatonin. The antibody against melatonin also displayed partial IR for N-acetyl serotonin (<0.01%). Hence, we cannot exclude the possibility of false positive melatonin IR in cells producing serotonin. This potential confounder was minimized through co-localization studies where the subcellular localizations for serotonin and melatonin only partially overlap (see Fig. 3A-C). Another indication that this finding is true was the presence of EC cells where IR to melatonin greatly exceeded that to serotonin.

A large number of EC cells throughout the GI tract also displayed MT_2_ IR. This is a different finding from that found in the rat intestine where MT_2_ immunolabeling was expressed predominantly within the muscular layers but not in the mucosa [26]. Co-localization studies with serotonin showed MT_2_ receptor IR to be present in the cytoplasm in separate locations. MT_1_ receptor IR was generally not seen in EC cells, which is possibly due to periodicity in MT_1_ receptor expression as described for MT_1_ in the brain, where cAMP levels stimulates MT_1_ up-regulation and melatonin stimulates MT_1_ down-regulation [47–50].

Melatonin is a fat-soluble compound that easily crosses cell membranes and the blood-brain barrier. Melatonin is not thought to be stored in cells upon production [18] but rather immediately released. In the intestine, high concentrations of luminal melatonin increase bicarbonate secretion in the duodenum in response to acidic luminal contents via the MT_2_ receptor, theoretically protecting the intestinal mucosa [35]. We were therefore expecting staining of MT_2_ in the distal duodenal mucosa but this was only found in few. One possible explanation for this may be that proton pump inhibitor medication is commonly administered pre-surgery in order to diminish the risk of aspiration. Thus, a reduced acid load on the mucosa may have affected the expression of duodenal melatonin.

Epithelial melatonin staining was strongest in the colon and rectum. These same sections also displayed very strong epithelial expression of both MT_1_ and MT_2_ receptors. The MT_1_ IR results are in agreement with findings previously described for human colonic mucosa [27]. Recently, it was shown that very high doses of melatonin reduce epithelial paracellular permeability in rats and may prevent deleterious substances such as endotoxins from leaking in and causing inflammation [51]. An alleviating role has been ascribed to melatonin in the elusive interplay between different aggressive and protective factors in experimental colitis [52–54]. We tested the hypothesis that melatonin receptors and enzymes needed for synthesis may be up-regulated in inflammatory bowel disease compared to controls but found no large differences in mRNA expression. We did note small but significant changes in TDO2 (logFC = 0.22, p<0.01), IDO1 (logFC = 0.38, p = 0.0004) and DDC (logFC = -0.19, p = 0.000004). The cumulative effect of these changes indicates a shift in the tryptophan metabolism towards the kynurenine pathway.

The relationship between GI melatonin and circulating levels in the plasma appears to be bidirectional. It has been demonstrated that circulating melatonin and ingested melatonin accumulate in the GI tract [55, 56]. In the gut, melatonin appears to act as a functional antagonist of serotonin [29] and dampens intestinal motility [30]. Accordingly, we found MT_1_ and MT_2_ IR in both the submucosal and myenteric plexuses, which have both parasympathetic and sympathetic input. In vascular structures of the gut, weak MT_1_ IR was seen in endothelial cells, whereas more distinct MT_2_ IR was found in vascular smooth muscle cells. In blood vessels, melatonin activation of MT_2_ causes vasodilation, while MT_1_ mediates vasoconstriction [57]. There is also evidence that melatonin regulates endothelial permeability permitting leukocyte extravasation in the course of an immune challenge [58]. In support of this, melatonin and the MT_2_ receptor were seen in mononuclear cells in the lamina propria. It has been suggested that lymphocytes produce melatonin, which, in an autocrine and paracrine manner, promotes IL-2 production and proliferation of both T-cells and macrophages [58].

Melatonin IR in pancreatic islets was a surprising finding. Generally low TPH1 expression and some individuals with elevated levels, indicates that serotonin production may be regulated on the RNA level in the pancreas. Gene expression of TDO2 and IDO1 were higher in the whole pancreas samples compared to the islets. This finding is supported by data in the Human Protein Atlas where TDO2 and IDO1 are expressed primarily in the exocrine pancreatic tissue (http://www.proteinatlas.org/ENSG00000151790-TDO2/tissue/pancreas) and (http://www.proteinatlas.org/ENSG00000131203-IDO1/tissue/pancreas).

Previous studies suggest that melatonin can be produced in pancreas. Gene expression of enzymes involved in the synthesis of melatonin, aralkylamine N-acetyltransferase and N-acetylserotonin O-methyltransferase, have been detected in rat pancreatic acinar cells [59] and human pancreas [60] giving support to our findings. It has also been shown that melatonin can influence transcription factors involved in insulin secretion in the pancreas in a receptor-dependent manner [61]. MT_1_ and MT_2_ were also demonstrated in pancreatic islets, which is in agreement with melatonin’s newly described role in regulating circulating glucose levels via insulin and glucagon secretion [1–3]. The MT_2_ IR was found to be the dominant expression, which is a different result than previous studies of mRNA expression in human islets where MT_1_ expression was suggested to be higher than MT_2_ [2]. Our analyses of pancreas gene expression in three separate data sets performed on two different platforms and our IHC results are in agreement that the MT_2_ expression levels exceed MT_1_. Notably, decreased melatonin secretion is reported to increase the risk of developing type 2 diabetes [62], as well as genetic variants of MT_2_ that lead to impaired melatonin signaling [63]. MT_1_ expression varied from strong to absent in all sections available, and the reason for this variation is not clear.

Our study demonstrates the presence of melatonin and its receptors, MT_1_ and MT_2,_ throughout the GI tract and in the pancreas. It seems that melatonin signaling may be autocrine, paracrine and/or endocrine and the multiple roles ascribed to it are dependent on organ localization and physiological context. Our results are in agreement with conceivable actions of melatonin, which include regulation of GI motility, epithelial protection, epithelial permeability, vascular function as well as entero-pancreatic endocrine cross-talk with impact on metabolic control. Elucidating the role of melatonin receptors and regulation of their expression may help in understanding the previously described association between disturbances in melatonin signaling in GI and metabolic diseases.

## Supporting Information

S1 Table Gene expression data for key enzymes in tryptophan serotonin and melatonin metabolism and receptors for melatonin and serotonin in small intestinal epithelium and pancreas. (DOCX)

S2 Table Gene expression data for key enzymes in tryptophan serotonin and melatonin metabolism and receptors for melatonin and serotonin in pancreatic islets. (DOCX)
