# Supplementary material for: Human Gastroenteropancreatic Expression of Melatonin and Its Receptors MT1 and MT2
Source: PLoS One. 2015 Mar 30;10(3):e0120195. doi: 10.1371/journal.pone.0120195 (PMC4378860; doi:10.1371/journal.pone.0120195)
Supplement: S1 Table — (DOCX) [file pone.0120195.s001.docx]

| **Gene Symbol** | **ProbesetID** | **GSE9576**  **Mean SD** | | **GSE16515**  **Mean SD** | | | **GSE15471**  **Mean SD** | |
| --- | --- | --- | --- | --- | --- | --- | --- | --- |
| TPH1 | 1553859_at | 3,1 | 0,3 | 3,3 | 0,4 | 3,7 | | 0,6 |
| TPH1 | 214601_at | 3,2 | 0,9 | 3,1 | 1,1 | 5,0 | | 1,2 |
| TPH2 | 1555332_at | 2,8 | 0,2 | 2,8 | 0,2 | 2,8 | | 0,1 |
| TDO2 | 205943_at | 5,4 | 1,9 | 4,6 | 1,9 | 4,6 | | 0,8 |
| TDO2 | 231702_at | 3,0 | 0,1 | 3,2 | 0,2 | 3,1 | | 0,2 |
| IDO1 | 210029_at | 6,3 | 0,7 | 6,4 | 1,1 | 7,8 | | 0,3 |
| IDO2 | 1568638_a_at | 3,2 | 0,2 | 3,5 | 0,5 | 2,9 | | 0,2 |
| DDC | 205311_at | 7,5 | 1,4 | 7,6 | 2,0 | 10,8 | | 0,2 |
| DDC | 214347_s_at | 5,1 | 0,9 | 5,5 | 1,2 | 8,2 | | 0,1 |
| AANAT | 207225_at | 3,3 | 0,3 | 3,5 | 0,3 | 3,4 | | 0,1 |
| ASMT | 206779_s_at | 3,6 | 0,2 | 3,8 | 0,3 | 3,7 | | 0,2 |
| ASMT | 210551_s_at | 3,7 | 0,3 | 3,9 | 0,3 | 4,2 | | 0,2 |
| ASMTL | 209394_at | 7,0 | 0,2 | 7,0 | 0,3 | 7,0 | | 0,1 |
| ASMTL | 36553_at | 8,1 | 0,5 | 7,7 | 0,4 | 8,2 | | 0,1 |
| ASMTL | 36554_at | 6,6 | 0,4 | 6,4 | 0,3 | 6,6 | | 0,1 |
| MAOA | 204388_s_at | 7,8 | 1,0 | 8,1 | 1,2 | 10,8 | | 0,3 |
| MAOA | 204389_at | 6,7 | 0,9 | 7,0 | 1,1 | 10,4 | | 0,1 |
| MAOA | 212741_at | 8,4 | 0,9 | 8,8 | 1,1 | 11,7 | | 0,1 |
| MTNR1A | 221369_at | 3,9 | 0,4 | 3,9 | 0,2 | 3,8 | | 0,1 |
| MTNR1B | 208516_at | 4,6 | 0,4 | 4,8 | 0,3 | 4,8 | | 0,1 |
| HTR1A | 221351_at | 4,9 | 0,4 | 5,1 | 0,5 | 4,8 | | 0,3 |
| HTR1B | 210799_at | 4,0 | 0,5 | 4,3 | 0,4 | 4,2 | | 0,3 |
| HTR1D | 207368_at | 3,7 | 0,4 | 4,2 | 0,5 | 4,2 | | 0,1 |
| HTR1E | 207404_s_at | 4,0 | 0,5 | 4,1 | 0,4 | 3,8 | | 0,2 |
| HTR1F | 221458_at | 3,6 | 0,3 | 3,6 | 0,3 | 3,4 | | 0,1 |
| HTR2A | 207135_at | 4,1 | 0,7 | 4,0 | 0,5 | 3,8 | | 0,0 |
| HTR2A | 211616_s_at | 3,6 | 0,5 | 3,5 | 0,3 | 3,2 | | 0,1 |
| HTR2B | 206638_at | 5,1 | 1,7 | 4,3 | 1,5 | 3,4 | | 0,2 |
| HTR2C | 207307_at | 2,7 | 0,1 | 2,6 | 0,1 | 2,7 | | 0,1 |
| HTR2C | 211479_s_at | 2,6 | 0,2 | 2,6 | 0,2 | 2,8 | | 0,1 |
| HTR3A | 216615_s_at | 4,7 | 0,5 | 4,7 | 0,3 | 4,6 | | 0,2 |
| HTR3A | 217002_s_at | 4,1 | 0,5 | 4,1 | 0,3 | 4,2 | | 0,3 |
| HTR3B | 221084_at | 3,6 | 0,5 | 4,1 | 0,4 | 3,9 | | 0,2 |
| HTR3C | 1553041_at | 4,8 | 0,7 | 5,1 | 0,7 | 4,9 | | 0,3 |
| HTR4 | 207577_at | 2,9 | 0,3 | 3,1 | 0,3 | 3,4 | | 0,4 |
| HTR4 | 207578_s_at | 6,9 | 0,4 | 7,1 | 0,4 | 6,9 | | 0,2 |
| HTR4 | 216939_s_at | 2,8 | 0,2 | 3,1 | 0,2 | 3,4 | | 0,2 |
| HTR5A | 221362_at | 4,8 | 0,3 | 5,0 | 0,4 | 4,8 | | 0,2 |
| HTR6 | 1552857_a_at | 4,1 | 0,4 | 4,2 | 0,4 | 4,3 | | 0,4 |
| HTR6 | 206944_at | 4,3 | 0,4 | 4,8 | 0,4 | 5,0 | | 0,1 |
| HTR7 | 207818_s_at | 3,1 | 0,2 | 3,2 | 0,3 | 3,2 | | 0,2 |
| HTR7 | 207927_at | 4,9 | 0,3 | 5,3 | 0,4 | 5,4 | | 0,2 |
| HTR7 | 236281_x_at | 3,0 | 0,1 | 3,2 | 0,2 | 3,6 | | 0,2 |
| PTH | 206977_at | 2,5 | 0,2 | 2,6 | 0,2 | 2,6 | | 0,1 |
