# Supplementary material for: Human Gastroenteropancreatic Expression of Melatonin and Its Receptors MT1 and MT2
Source: PLoS One. 2015 Mar 30;10(3):e0120195. doi: 10.1371/journal.pone.0120195 (PMC4378860; doi:10.1371/journal.pone.0120195)
Supplement: S2 Table — (DOCX) [file pone.0120195.s002.docx]

| Gene Symbol | ProbesetID | GSE38642  Mean SD | |
| --- | --- | --- | --- |
| TPH1 | 7946946 | 4,3 | 0,6 |
| TDO2 | 8097991 | 4,2 | 0,7 |
| IDO1 | 8146092 | 4,5 | 0,7 |
| IDO2 | 8146105 | 3,4 | 0,2 |
| DDC | 8139640 | 6,6 | 0,5 |
| AANAT | 8010071 | 5,3 | 0,2 |
| ASMTL | 8171119 | 7,1 | 0,2 |
| ASMTL | 8177011 | 7,1 | 0,2 |
| MAOA | 8166925 | 7,6 | 0,4 |
| MTNR1A | 8104074 | 5,4 | 0,4 |
| MTNR1B | 7943098 | 5,6 | 0,2 |
| HTR1A | 8112333 | 5,1 | 0,3 |
| HTR1B | 8127692 | 5,9 | 0,3 |
| HTR1D | 7913566 | 5,1 | 0,4 |
| HTR1E | 8120983 | 3,6 | 0,2 |
| HTR1F | 8081067 | 6,3 | 0,6 |
| HTR2A | 7971526 | 3,6 | 0,3 |
| HTR2B | 8059680 | 3,4 | 0,3 |
| HTR2C | 8169447 | 4,2 | 0,2 |
| HTR3A | 7943969 | 6,5 | 0,5 |
| HTR3B | 7943958 | 3,1 | 0,2 |
| HTR3C | 8084275 | 4,3 | 0,2 |
| HTR5A | 8137517 | 4,9 | 0,2 |
| HTR6 | 7898594 | 5,3 | 0,2 |
| HTR7 | 7934970 | 4,9 | 0,2 |
| PTH | 7946687 | 2,5 | 0,2 |
